# Tuberculosis-Induced Bronchiectasis Complicated by Recurrent Respiratory Tract Infections and Renal Amyloidosis: A Classic Revisited

**DOI:** 10.7759/cureus.11638

**Published:** 2020-11-23

**Authors:** Ayesha Akram

**Affiliations:** 1 Internal Medicine, Rawalpindi Medical University, Rawalpindi, PAK

**Keywords:** pulmonary tuberculosis, treated, complication, bronchiectasis, renal amyloidosis

## Abstract

In this case, a young male patient with a past medical history of adequately treated pulmonary tuberculosis (TB), presented with pedal edema, proteinuria, and evidence of bilaterally enlarged kidneys on renal ultrasound, raising suspicion of renal amyloidosis. Cough, expectoration, severe dyspnea, and high-resolution computed tomographic changes of dilated bronchi paralleled evidence of bronchiectasis exacerbated by perpetual bacterial infection. In view of the laboratory findings and imaging studies, a renal biopsy was done, and it supported the diagnosis of secondary amyloidosis in the kidneys. Clearly, TB infection, although treated, had exerted a multifaceted effect, and it ran a downward spiral from there: the simultaneous occurrence of bronchiectasis and recurrent respiratory tract infections, renal amyloidosis, nephrotic syndrome and an inevitable end-stage renal failure in just the third decade of life. It makes sense then, to use adjuvant steroid therapy as complementing traditional TB therapy to combat the destructive and fibrosing properties of pulmonary TB.

## Introduction

Among the many varied causes of bronchiectasis, necrotizing infection is a common cause. The infectious agents commonly implicated are bacterial (*Haemophilus influenzae* and *Pseudomonas aeruginosa*) and granulomatous (*Mycobacterium tuberculosis* and atypical mycobacteria) [1]. Bronchiectasis as a sequela of pulmonary tuberculosis (TB) is certainly not rare and can persist or worsen despite TB treatment completion [2]. In turn, bronchiectatic airways are often colonized with bacterial species. When a chronic inflammatory condition such as TB or bronchiectasis leads to amyloidosis, the disorder is considered secondary amyloidosis [3]. Bronchiectasis and TB are a frequent association, but subsequent renal amyloidosis and end-stage renal disease (ESRD) was a combination much less frequent in my literature search, with a mean duration of bronchiectasis hovering around 22 years [4]. I present a case of renal amyloidosis secondary to post-tuberculous bronchiectasis, presenting after a latent period of 16 years.

## Case presentation

A 32-year-old male was admitted to the medicine ward with chief complaints of generalized body swelling and decreased urine output since two months. The feet and legs had swollen first, and then the face and periorbits. Six months back, the patient first began to go down-hill. He developed dyspnea unrelated to exertion and a cough associated with daily mucopurulent, occasionally blood-streaked, foul-smelling sputum. He was fatigued most days and nights, and a low-grade fever had been present intermittently, but he reported no weight loss, wheezing or pleuritic chest pain. In retrospect, he was diagnosed with pulmonary TB in February, 2004 on the basis of sputum smear microscopy for acid-fast bacilli (AFB) and history of contact with a patient of active TB, received appropriate antitubercular therapy (ATT) for it; Tab Myrin-P Forte 4×once daily (OD) for two months, followed by Tab Myrin 4×OD for four months and Tab Vita-6 1×OD for six months; and recovered thereafter. Following that, his temperature was never over 99 F. Sputum was positive until July, 2004, and was said to have been negative after that time. He had no history of any other pulmonary illness, diabetes, or hypertension. On examination at arrival he was nauseated, pale in the conjunctivae, febrile with an axillary temperature of 38.4 C, hypotensive to 70/40 mmHg, tachycardic with a pulse rate of 104 beats/min, slightly tachypneic at a respiratory rate of 24 breaths/min, and showed signs of periorbital edema, facial puffiness, and lower extremity pitting edema. The lungs were dull to percussion, a few rhonchi over the right lung and diffuse, bilateral crackles were heard on auscultation. The infection protocol was implemented, including intravenous crystalloids, empiric broad-spectrum antibiotics, and chest physiotherapy. There were no signs of joint swelling, any rash or discoloration, or evidence of heart failure or enlargement. Early laboratory investigations revealed: hemoglobin, 6.9 g/dL with normal mean corpuscular volume; leukocyte count, 12.5 × 10^9^/L; platelet count, 388 × 10^9^/L; serum albumin, 2.0 g/dL; serum calcium, 6.7 mg/dL; serum potassium, 5.9 mEq/L; a markedly elevated serum creatinine at 271 μmol/L and an elevated serum urea at 14.3 mmol/L. Parathyroid hormone level was 146.9 pg/mL (normal: 10-65 pg/mL). Urinalysis showed protein (++), hyaline casts, and 24-hour urine protein raised at 3.7 g/day. Serum immunoglobulin levels were normal thus ruling out common variable immunodeficiency as a cause of secondary amyloidosis. Table 1 gives an overview of the investigations. He was started on angiotensin-converting enzyme inhibitors, and a one-off dialysis session after nephrology consultation.

**Table 1:** An analysis of the laboratory results MCV, mean corpuscular volume; PTH, parathyroid hormone

Laboratory test results	Presumptive diagnosis
Proteinuria, hypoalbuminemia and resulting pitting edema	The patient was diagnosed as a case of nephrotic syndrome secondary to ?
Hyperkalemia, low hemoglobin with normal MCV	Hyperkalemia and a normocytic anemia were taken as manifestations of chronic renal failure
Hypocalcemia, elevated PTH	Secondary hyperparathyroidism, yet another manifestation of chronic renal failure

**Figure 1:**
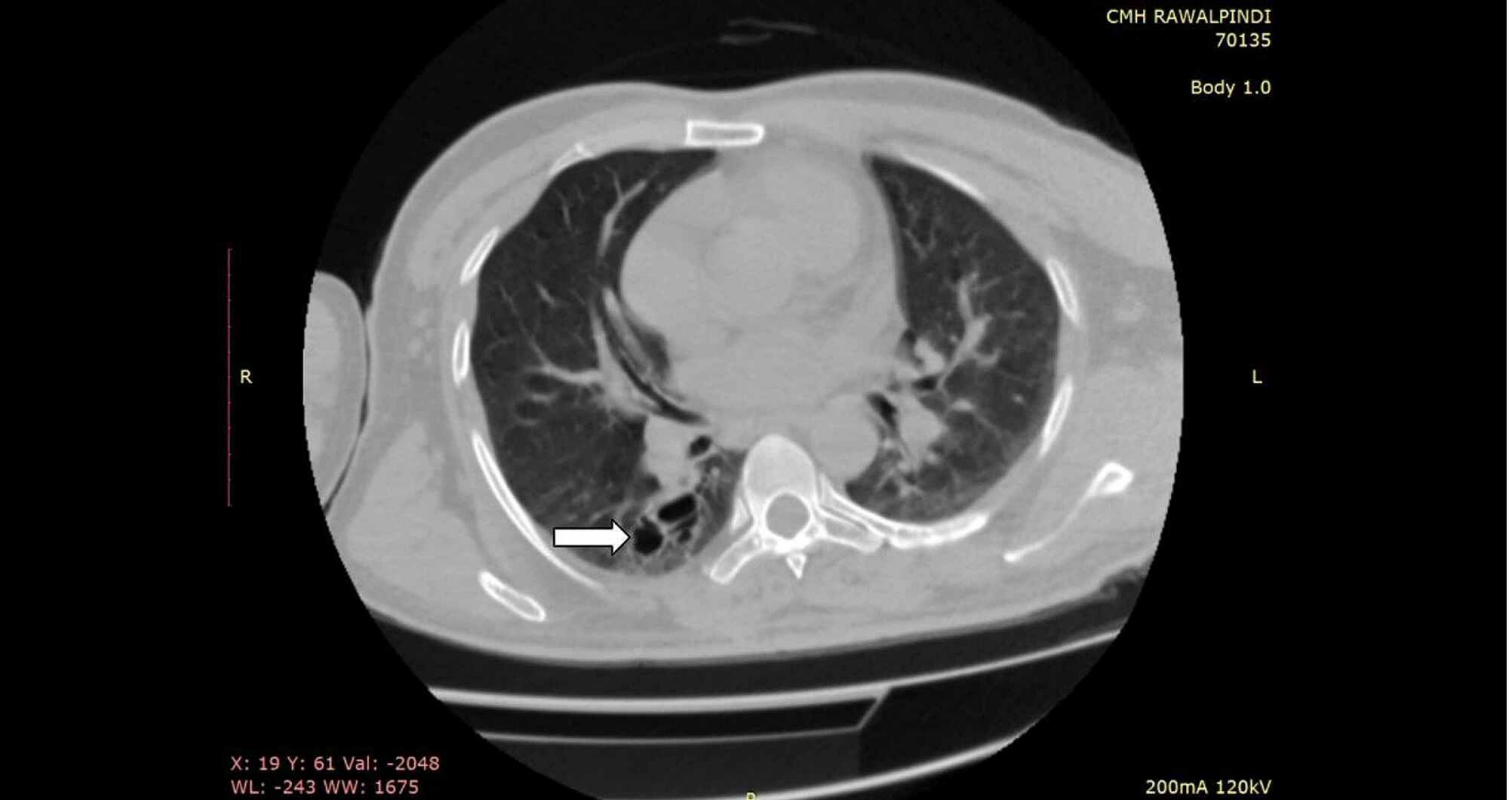
Axial thoracic HRCT shows a moderately bronchiectatic right lung, considered sequelae of previous tuberculosis cystic bronchiectasis in the superior segment of the right lower lobe (arrow). HRCT, high-resolution computed tomography

**Figure 2:**
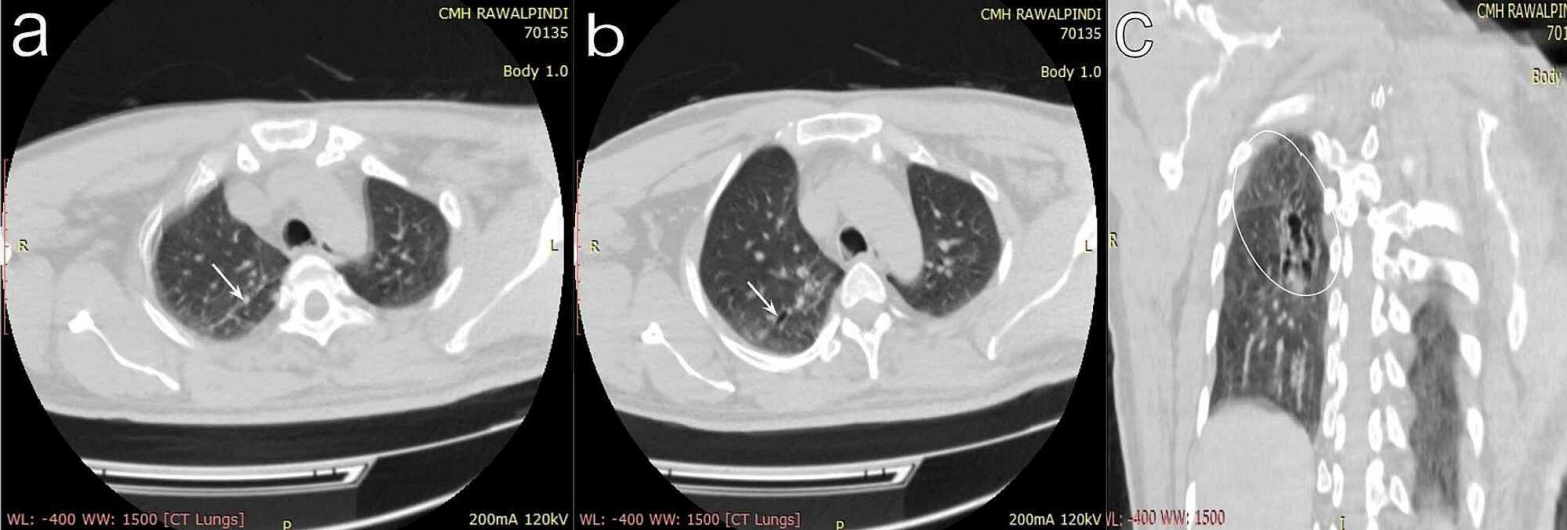
Thoracic HRCT shows a moderately bronchiectatic right lung attributable to old resolved tuberculosis a: a fibrotic band in the apical segment of the right upper lobe (arrow) on axial view. b: fibrosis and traction bronchiectasis (arrow) in the posterior segment of the right upper lobe on axial view. c: fibrosis and tubular bronchiectasis in the superior segment of the right lower lobe (circle) on coronal view. HRCT, high-resolution computed tomography

A single sputum smear was negative for AFB. A high-resolution computed tomography scan done about four months prior was consistent with bronchiectasis in the right lung, with no evidence of active TB (Figures 1, 2).

**Figure 3:**
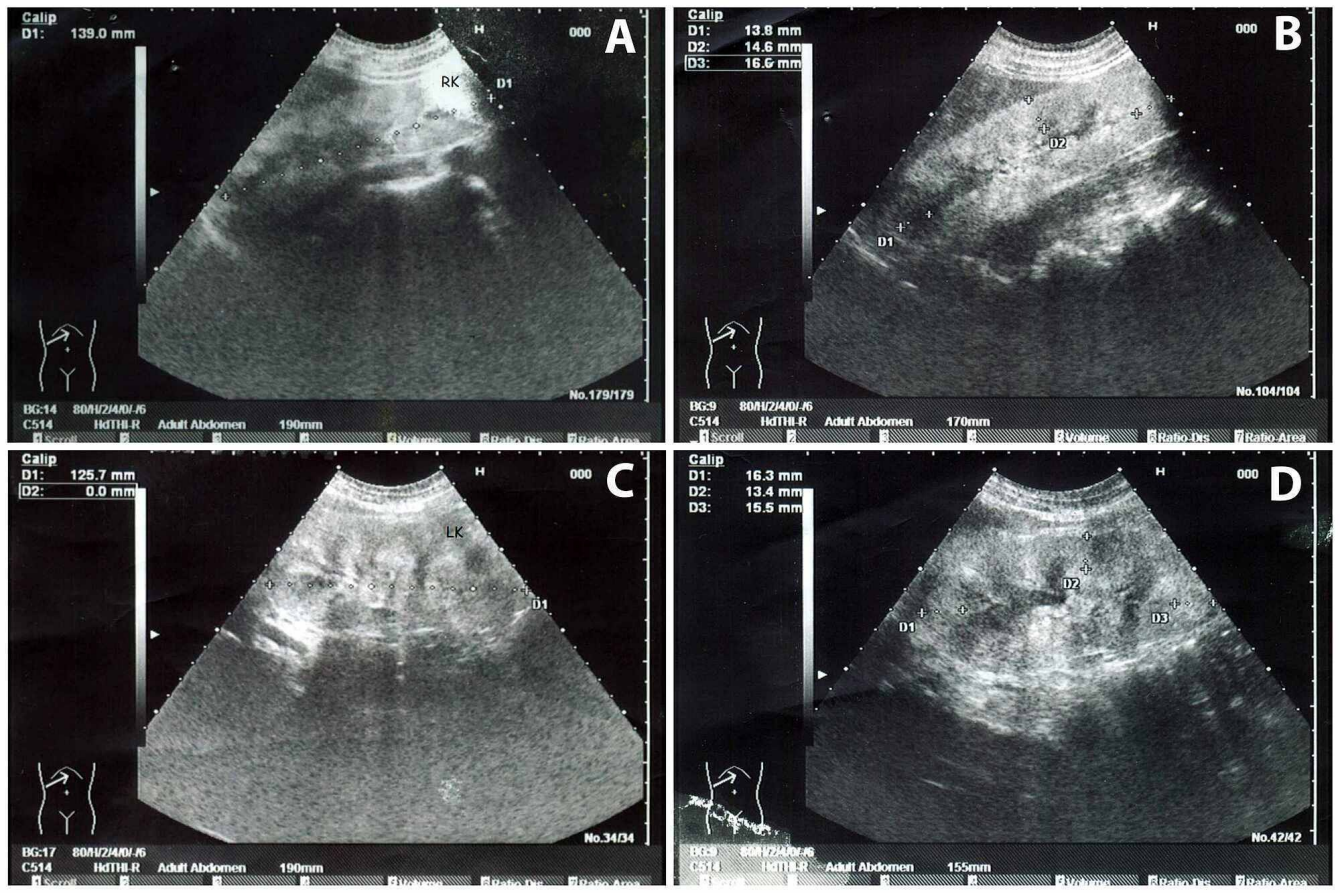
Bilateral enlarged, echogenic kidneys with Grade II renal parenchymal changes on renal ultrasound A: Right kidney enlarged in size measuring approximately 13.9 cm, with increased parenchymal echogenicity and poor corticomedullary differentiation. Measurement of kidney length is illustrated by ‘+’, a dashed line and D1, RK indicating right kidney. B: Renal cortical parenchymal thickness of right kidney measured 13.8 mm at upper pole (D1), 14.6 mm at mid pole (D2), and 16.6 mm at lower pole (D3). C: Left kidney mildly enlarged in size measuring approximately 12.6 cm, with increased parenchymal echogenicity and poor corticomedullary differentiation. Measurement of kidney length is illustrated by ‘+’, a dashed line and D1, LK indicating left kidney. D: Renal cortical parenchymal thickness of left kidney measured 16.3 mm at upper pole (D1), 13.4 mm at mid pole (D2), and 15.5 mm at lower pole (D3).

Ultrasonography of the kidneys revealed bilaterally enlarged, echogenic kidneys with poor corticomedullary differentiation suggesting chronic kidney disease (Figure 3).

**Figure 4:**
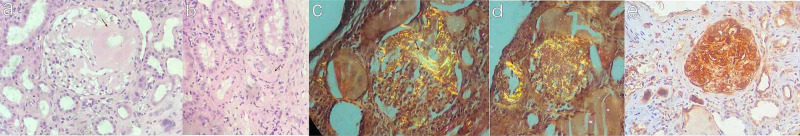
Morphological and immunohistochemical features consistent with secondary amyloidosis on renal biopsy a: amorphous eosinophilic PAS-positive deposits in the glomerular mesangium (arrow) on H&E stain. b: arterioles showed amorphous eosinophilic PAS-positive deposits (arrow) on H&E stain. c, d: apple-green birefringence in the glomeruli and arterioles (arrows) after staining with Congo red, viewed under polarized light. e: Amyloid A positive on immunohistochemistry. H&E, hematoxylin and eosin; PAS, periodic acid-Schiff

Suspecting amyloidosis, a biopsy was in order. The biopsy came directly from the kidneys, revealing renal amyloidosis (Figure 4).

Soon the symptoms became worse. By the third inpatient day, he ran a daily fever, continued to have a productive cough, raising from one-quarter to one cupful of sputum a day, and became hypoxic to the extent of utilizing 3 L per minute oxygen by nasal cannula. The third day of hospitalization saw a sizable drop in blood pressure to 50/35 mmHg, an increase in heart rate to 151 beats/min, and more rapid breathing at 30/min. Norepinephrine, vasopressin over titrating norepinephrine dose, and phenylephrine were used as a resort. However, the combination of all three vasopressors had little further effect. He was unable to maintain a mean arterial pressure above 65 mmHg, and breathed his last during the day.

## Discussion

Bronchiectasis, an irreversible dilatation of the bronchi and bronchioles, is a vicious cycle that starts with a chronically inflamed wall. The necrotizing inflammation has an adverse effect on mucociliary clearance and airway patency, rendering the under-protected bronchi susceptible to secondary bacterial invaders [1]. The effect of traction fibrosis in producing bronchial dilatation by the action of retracting scar tissue pulling on the bronchial wall has also long been recognized [2]. In this case, radiologic evidence suggests bronchiectatic involvement of the posterior and superior segment of the upper lobe and lower lobe of the right lung, respectively, putting TB back in the news many years later. The malignancy of the collusion is, multiple inflammatory risk factors often converge, amplifying the risk of developing systemic amyloidosis too. Serum amyloid A, an acute-phase reactant that increases in chronic inflammatory states, microbial infections and rarely, malignancy, deposits extracellularly in various organs including the kidney as insoluble fibrils to cause amyloid A (AA) secondary amyloidosis [5]. Renal amyloidosis itself reflects a poor prognosis. Dialysis, transplantation, or renal allograft are associated with poor outcomes. In fact, disease recurrence in the allograft may occur, and the risk of dying by amyloidosis in ESRD is high [6]. This and another case in literature are compared below; although the trends differ slightly, they are similar in prevalence of TB (Table 2). This is a stark illustration of a deadly pattern.

**Table 2:** A like-for-like comparison M, male; CVID, common variable immunodeficiency; TB, tuberculosis; AFB, acid-fast bacilli; ATT, antitubercular treatment; CT, computed tomography; HRCT, high-resolution computed tomography; AA amyloidosis, amyloid A amyloidosis

Reference		[7]	This case
Publication journal/year		Journal of Nephropharmacology/2015 [7]	Cureus/2020
Age/gender		40 y/M	32 y/M
Comorbid		CVID	None
TB detected		Sputum smear-positive for AFB	Sputum smear-positive for AFB
Started on ATT		Isoniazid, rifampicin, ethambutol and pyrazinamide for 3 months, followed by isoniazid and rifampicin for 6 more months	Isoniazid, rifampicin, ethambutol, and pyrazinamide for 2 months, followed by ethambutol, isoniazid and rifampicin for 4 months
Treatment success or failure		Success. Old healed TB	Success. Old healed TB
Thoracic CT/HRCT findings		Bronchiectatic changes with fibrosis in upper lobes of both lungs attributable to TB	Bronchiectatic changes with fibrosis in the right lower and upper lobes attributable to TB
Renal biopsy findings		AA amyloidosis	AA amyloidosis
Ultrasonography findings		Bilaterally enlarged kidneys with normal corticomedullary differentiation	Bilaterally enlarged kidneys with poor corticomedullary differentiation
Inflammation fueled by?		Additive effects of TB and recurrent respiratory tract infections due to CVID	TB and recurrent respiratory tract infections due to bronchiectasis only
Progress from TB to renal amyloidosis (years)		8	16
Laboratory investigations relevant to nephrotic syndrome	24-hour urine protein (g/day)	3.4	3.7
	Serum albumin (g/dL)	1.4	2.0
Patient outcome		Discharged on symptomatic treatment and an angiotensin receptor blocker. However, no details on follow-up	Fatal due to septicemia and hypoxemia

The premise is that, if patients with TB are cured, mortality will disappear, and prevalence will decline. The reality, however, is that TB is best understood as a spectrum. Granulomas - that is, infiltrating macrophages and lymphocytes - wall off the infection, although this is only partially protective. The interactions between the pathogen and the person’s immune response facilitate granulomatous inflammation with tissue damage, for example, caseation, fibrosis, and bronchiectasis [2]. This phenomenon of bronchiectasis, often long after infection or after treatment, indicates the challenge. Hence, immunosuppressive and anti-inflammatory drugs such as steroids, new preventive strategies, and more effective interventions will be required to tackle pulmonary impairment after TB, while assessing their risk-benefit ratios [2].

## Conclusions

Turning the tide on TB will require early and accurate case detection, and, where possible, preventive treatment of primary TB. The prevalence of bronchiectasis and resultant productive cough in treated pulmonary TB is high, and waiting for patients to become sick enough to seek care does not solve the problem. Secondary amyloidosis itself can only be prevented by promptly treating or preventing the inflammatory disease that triggered amyloidosis. For example, preventing TB or treating airway infections should stop secondary amyloidosis from getting worse. I conclude with advice on: a vaccine to prevent infection by *Mycobacterium tuberculosis*, new drugs, and treatment regimens for active TB and latent TB infection.
